# Ablation of ceramide synthase 2 exacerbates dextran sodium sulphate‐induced colitis in mice due to increased intestinal permeability

**DOI:** 10.1111/jcmm.13267

**Published:** 2017-07-12

**Authors:** Ye‐Ryung Kim, Giora Volpert, Kyong‐Oh Shin, So‐Yeon Kim, Sun‐Hye Shin, Younghay Lee, Sun Hee Sung, Yong‐Moon Lee, Jung‐Hyuck Ahn, Yael Pewzner‐Jung, Woo‐Jae Park, Anthony H. Futerman, Joo‐Won Park

**Affiliations:** ^1^ Department of Biochemistry College of Medicine Ewha Womans University Seoul South Korea; ^2^ Department of Biomolecular Sciences Weizmann Institute of Science Rehovot Israel; ^3^ College of Pharmacy Chungbuk National University Chongju South Korea; ^4^ Department of pathology College of Medicine Ewha Womans University Seoul South Korea; ^5^ Department of Biochemistry School of Medicine Gachon University Incheon South Korea

**Keywords:** inflammatory bowel disease, ceramide, sphingolipid, acyl chains, dextran sodium sulphate

## Abstract

Ceramides mediate crucial cellular processes including cell death and inflammation and have recently been implicated in inflammatory bowel disease. Ceramides consist of a sphingoid long‐chain base to which fatty acids of various length can be attached. We now investigate the effect of alerting the ceramide acyl chain length on a mouse model of colitis. Ceramide synthase (CerS) 2 null mice, which lack very‐long acyl chain ceramides with concomitant increase of long chain bases and C16‐ceramides, were more susceptible to dextran sodium sulphate‐induced colitis, and their survival rate was markedly decreased compared with that of wild‐type littermates. Using mixed bone‐marrow chimeric mice, we showed that the host environment is primarily responsible for intestinal barrier dysfunction and increased intestinal permeability. In the colon of CerS2 null mice, the expression of junctional adhesion molecule‐A was markedly decreased and the phosphorylation of myosin light chain 2 was increased. *In vitro* experiments using Caco‐2 cells also confirmed an important role of CerS2 in maintaining epithelial barrier function; CerS2‐knockdown *via* CRISPR‐Cas9 technology impaired barrier function. *In vivo* myriocin administration, which normalized long‐chain bases and C16‐ceramides of the colon of CerS2 null mice, increased intestinal permeability as measured by serum FITC‐dextran levels, indicating that altered SLs including deficiency of very‐long‐chain ceramides are critical for epithelial barrier function. In conclusion, deficiency of CerS2 influences intestinal barrier function and the severity of experimental colitis and may represent a potential mechanism for inflammatory bowel disease pathogenesis.

## Introduction

Inflammatory bowel disease (IBD), which includes Crohn's disease and ulcerative colitis, is a chronic, relapsing, inflammatory disorder of the gastrointestinal tract resulting in abdominal pain, diarrhoea, rectal bleeding and weight loss 1. The prevalence of IBD is increasing worldwide, and it is considered an emerging global disease 2. The aetiology of IBD involves a complex interaction between genetic susceptibility and environmental risk factors (such as smoking, antibiotics, infection and oral contraceptives 2, 3). However, none of these factors are clear determinants of IBD, with inconsistent observations reported among various studies. Therefore, additional studies are needed to better understand the aetiology and pathogenesis of IBD.

Recently, sphingolipids (SLs) have emerged as targets for IBD therapy 1 *via* the roles they play in regulating immune cell function 4, 5 and mucosal integrity 6. For example, dextran sodium sulphate (DSS)‐induced experimental colitis is aggravated in animals lacking sphingosine 1‐phosphate lyase, the enzyme that degrades sphingosine‐1‐phosphate (S1P), whereas the severity of experimental colitis is diminished upon genetic knockout of sphingosine kinase 1 7. In addition, the administration of S1P antagonists diminishes the severity of experimental murine colitis 4, 5, 8. The role of S1P in IBD may be due to its regulation of CD4^+^ T cell function (such as migration and differentiation) or by activation of NF‐κB and STAT3 signalling 4, 5, 8, 9. Ceramide is a key player in the pathway of SL metabolism, and ceramide regulates a plethora of cellular responses, including cell proliferation, apoptosis, differentiation and senescence 10. Ceramide consists of a long‐chain base linked by an amide bonds to a fatty acid; the length of the fatty acid varies significantly.

The ceramide acyl chains length is determined by a family of six ceramide synthases (CerS) 11; CerS5/6 generates long‐chain (C14–C16‐) ceramides 11, 12, CerS1 generates C18‐ceramide 13, CerS2 generates very‐long chain (C22–C24‐) ceramides 14. The distinct role of ceramide depends on its acyl chain length, as shown in the various CerS‐deficient mice which have been generated over the past few years 15. For example, CerS1 deficiency results in Purkinje cell death and neuronal degeneration in the cerebellum 16, 17, whereas CerS2 deficiency results in a progressive demyelinating phenotype 18. Similarly, CerS2 deficiency results in insulin resistance 19, 20, whereas CerS5 or CerS6 deficiency leads to improved glucose homoeostasis 21, 22. However, the role of ceramides with distinct acyl chain lengths in IBD has not been studied.

In this study, we used CerS2 null mouse to investigate the role of very‐long chain ceramides in experimental colitis. In the colon of CerS2 null mice, levels of long‐chain bases and C16‐ceramides were elevated along with decrease of very‐long chain ceramides. CerS2 null mice exhibited severe colitis upon DSS treatment, along with increased intestinal permeability, higher myosin light chain 2 (MLC2) phosphorylation and diminished levels of junctional adhesion molecule‐A (JAM‐A) in the colon. *In vitro* experiments using Caco‐2 cells also indicated that CerS2 is crucial for maintaining epithelial barrier function. We suggest that CerS2, which regulates the levels of very‐long chain ceramides, is important for intestinal barrier function, and altered acyl chain length of ceramides might be involved in the pathogenesis of IBD.

## Materials and methods

### Animals

CerS2‐null mice have been described previously 23, 24. Mice, 6–10 week of age, were housed under special pathogen‐free conditions at 21–23°C and 51–54% humidity with a 12‐hr light–dark cycle and were supplied with food and water *ad libitum*. All animals were treated in accordance with the Animal Care Guidelines of Ewha Womans University and Weizmann Institute of Science, and the animal experiments and procedures were approved by the animal ethics committees at Ewha Womans University College of Medicine and the Weizmann Institute of Science.

### DSS‐induced colitis

**Figure 1:**
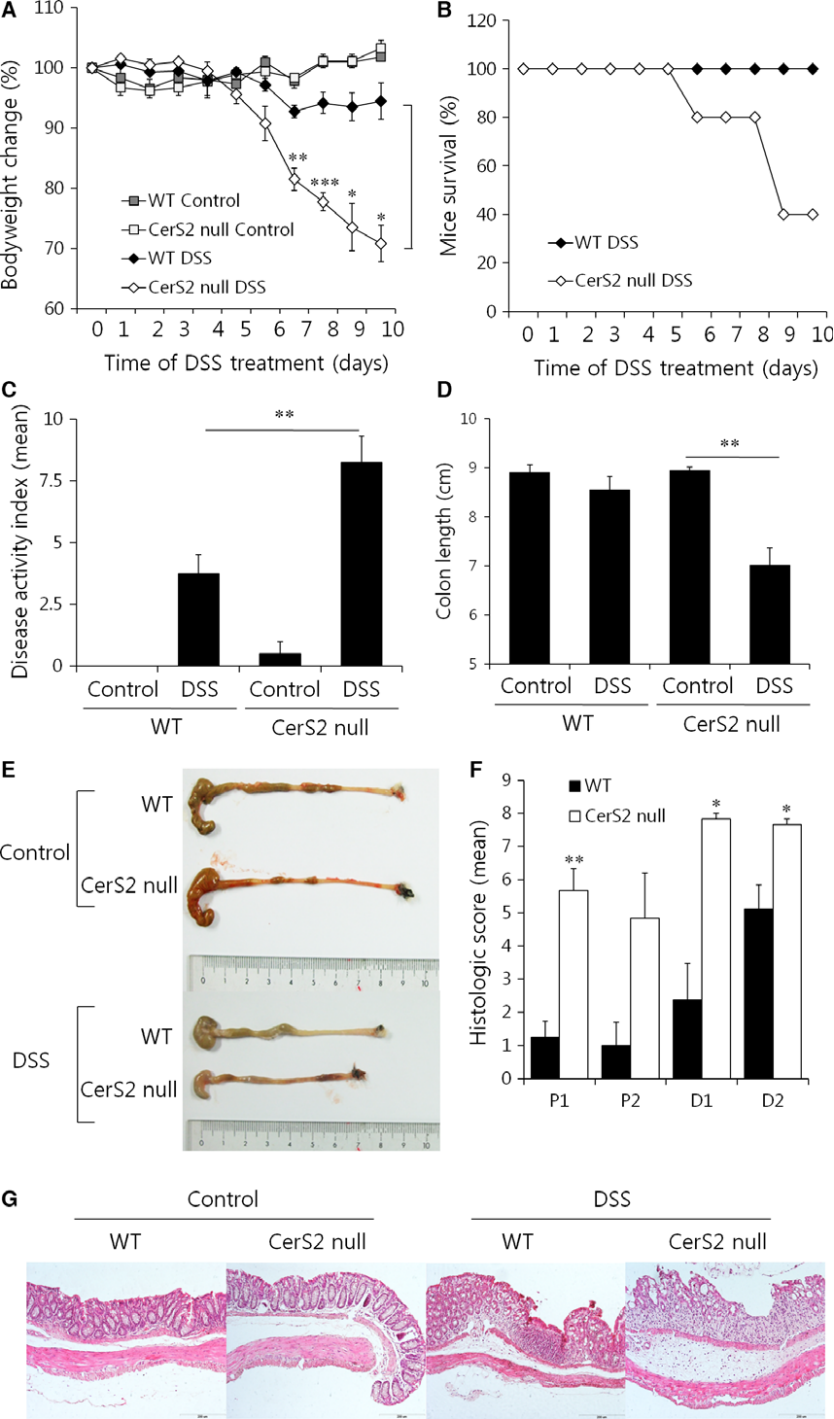
DSS‐induced colitis is exacerbated upon CerS2 deficiency. To induce colitis, mice were exposed to 3% (w/v) DSS in the drinking water for 5 days followed by normal plain water for another 5 days. For controls, normal plain water was consumed for 10 days. (**A**) Bodyweight changes and (**B**) survival curves of WT and CerS2 null mice. (**C**) Disease activity index values, (**D**) colon lengths, (**E**) representative colon pictures, (**F**) histological scores and (**G**) H & E‐stained colonic sections of mice. The scale bar indicates 200 μm. Data are means ± S.E.M. (*n* = 5). *P < 0.05, **P < 0.01, ***P < 0.001.

To evaluate the severity and mortality by murine colitis, DSS was first administered orally to mice in drinking water (3%, w/v) for 5 days, followed by 5 days of plain water consumption (see Fig. 1). For all other experiments, 2% (w/v) DSS was mixed in drinking water, and mice were sacrificed after 7 days of DSS treatment. Bodyweights were assessed daily during experiments, and a disease activity index (DAI) score was calculated to evaluate the clinical progression of colitis. The DAI is a combined score of weight loss compared to initial weight (0, no loss; 1, 1–5%; 2, 5–10%; 3, 10–20%; and 4, >20% loss), stool consistency (0, normal; 2, loose stool; and 4, diarrhoea) and rectal bleeding (0, no blood; 1, Hemoccult positive; 2, Hemoccult positive and visual pellet bleeding; and 4, gross bleeding, blood around anus) 25.

### Cell culture and generation of CerS2‐knockdown cells

Caco‐2 cells were cultured in Dulbecco's modified Eagle's medium (Hyclone, Logan, UT, USA) supplemented with 10% foetal bovine serum and 1% penicillin/streptomycin (Hyclone).

The *CERS2* gene was deleted from Caco‐2 cells using the CRISPR/Cas9 genome editing system and the GeneArt CRISPR nuclease vector with a CD4 enrichment kit (Invitrogen, Carlsbad, CA, USA) according to the manufacturer's protocol 26. Briefly, target‐specific oligonucleotides (top strand, 5′‐GCCGATCTAGAAGACCGAGAGTTTT‐3′; and bottom strand, 5′‐GATCGGTCTTCTAGATCGGTCGGTG‐3′) were synthesized by Macrogen (Seoul, South Korea), and a double‐stranded oligonucleotide with compatible ends was cloned into the GeneArt CRISPR nuclease vector. The presence of the double‐stranded oligonucleotide insert was confirmed by sequencing. Caco‐2 cells were transfected with the CRISPR/Cas9 vector using Metafectene (Biotex Laboratories, Edmonton, Alberta, Canada) according to the manufacturer's instructions. Transfected cells were screened and sorted using the CD4^+^ T cell isolation kit (Miltenyi Biotech, Bergisch Gladbach, Germany).

### Transepithelial electrical resistance

A transepithelial electrical resistance (TEER) assay was performed as reported previously 27. Briefly, Caco‐2 cells were seeded on tissue culture polycarbonate membrane filters (pore size, 0.4 μm) in 24‐well transwell plates at a seeding density of 2  ×  10^5^ cells/cm^2^ (Corning, Inc., Acton, MA, USA). The culture medium was added to the upper and lower chambers and was changed every second day. The cells were maintained to differentiate for 14 days after seeding with monitoring of TEER. Prior to TEER measurement, the standard medium was substituted with foetal bovine serum‐free culture medium. The electrical resistance was measured using a Millicell ERS meter (Millipore, Bedford, MA, USA) and calculated as Ω cm^2^.

### Statistical analyses

All the experiments were repeated at least three times independently, and values are given as means ± S.E.M. Statistical significance was calculated using Student's *t‐*tests, and *P* values of <0.05 were considered significant.

Details of other experiments are described in the supplementary information.

## Results

### CerS2 deficiency exacerbates DSS‐induced colitis

To investigate the role of the SL acyl chain length on IBD, colitis was induced by treating CerS2 null mice with DSS, a chemical irritant, due to its simplicity and many similarities with human ulcerative colitis 28. DSS was orally administered to mice in drinking water (3%, w/v) for 5 days, followed by 5 days of plain water consumption. Compared to WT controls, CerS2 null mice displayed aggravated colitis as manifested by greater bodyweight loss and worse survival rates (Fig. 1A and B). Disease activity index (DAI) values calculated by summing the scores reflecting weight loss, stool consistency and rectal bleeding were significantly elevated in CerS2 null mice (Fig. 1C). In addition, the length of the colons after DSS treatment was shorter in CerS2 null mice (Fig. 1D and E). The colons of untreated CerS2 null mice had a normal histological appearance, but higher scores of colonic epithelial damage and inflammatory infiltration were observed after DSS treatment (Fig. 1F and G). Similar results with no mortality were obtained by administration of 2% (w/v) DSS mixed in drinking water for 7 days (Fig. S1). Although the increase in blood leucocyte levels upon DSS treatment was similar in CerS2 null and WT mice, a greater increase in levels of neutrophils was observed in CerS2 null mice (Fig. S2). Together, these findings suggest that CerS2 deficiency exacerbates experimental colitis in mice.

### Ceramide acyl chain length in the colon of CerS2 null mice

**Figure 2:**
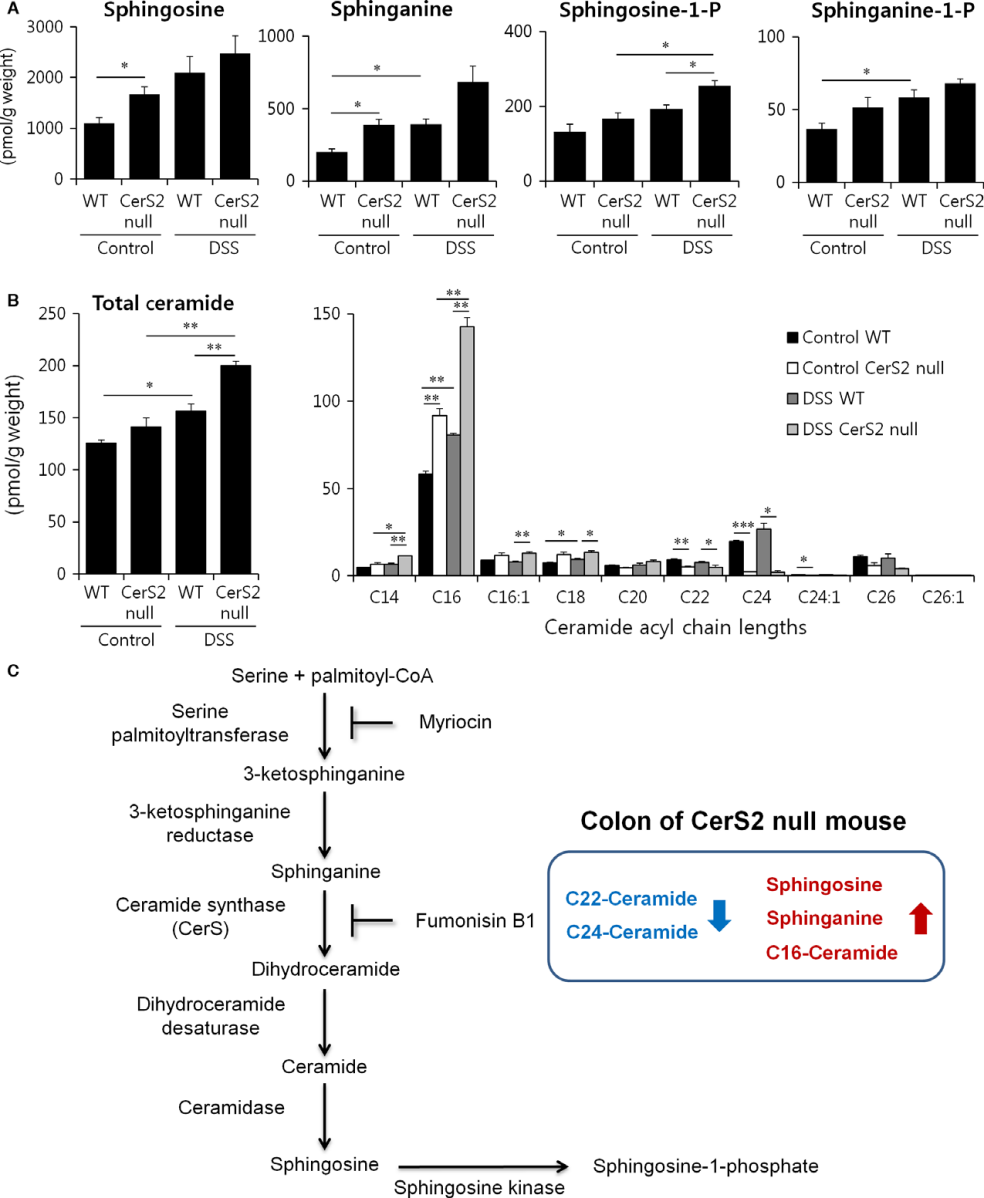
Effects of CerS2 deletion on colon sphingolipid levels upon DSS administration. Mice were exposed to 2% (w/v) DSS in the drinking water for 7 days to induce colitis or normal plain water (control). Long‐chain bases (**A**) and ceramides (**B**) were measured by ESI‐MS/MS. The *x* axis of the right panel in **B** shows the acyl chain lengths of the individual ceramide species. Results are expressed as means ± S.E.M. (*n* = 3). *P < 0.05, **P < 0.01, ***P < 0.001. (**C**) Scheme of SL metabolism and altered SL levels in the colon of CerS2 null mice were described.

Significant changes were detected in SL levels in the colon of CerS2 null mice. Similar to the SL composition in liver reported earlier 23, levels of long‐chain bases, including sphingosine and sphinganine, and C16‐ceramides were significantly elevated with concomitant decrease of C22‐C24 ceramides in the colon of CerS2 null mice (Fig. 2A and B), and total ceramide levels were not different between CerS2 null and WT mice (Fig. 2B), which is also similar to the liver 23. However, DSS administration in both WT and CerS2 null mice increased total ceramide levels as well as levels of long chain bases, which suggests activation of the *de novo* SL synthesis pathway (Fig. 2A and B). The elevated levels of total ceramides after DSS treatment were mainly derived from C16‐ceramide (Fig. 2B). Scheme of SL metabolism and altered SL levels in the colon of CerS2 null mice were described (Fig. 2C).

### The host environment plays a major role in the development of colitis in CerS2 null mice

**Figure 3:**
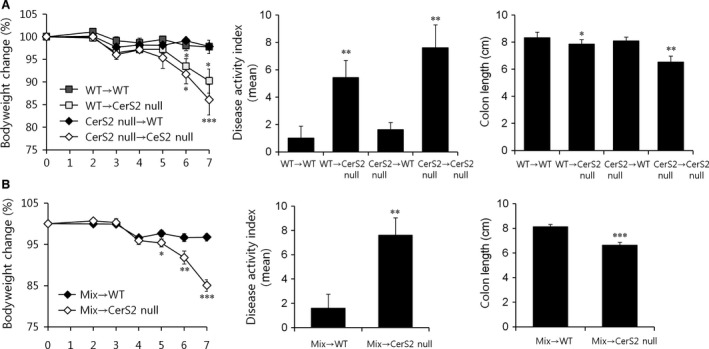
CerS2‐deficient host environments influence colitis severity in CerS2 null mice. Acute colitis was induced with 2% DSS in the drinking water for 7 days in (**A**) BM chimeras generated by injecting lethally irradiated WT or CerS2 null mice with 100% WT or CerS2 null BM cells and (**B**) mixed BM chimeras generated by injecting lethally irradiated WT or CerS2 null mice with 50% WT + 50% CerS2 null BM cells. Bodyweight changes were monitored during the experiment, and disease activity index scores and colon length were determined. Data are means ± S.E.M. (*n* = 3). *P < 0.05, **P < 0.01, ***P < 0.001. Mix, 50% WT + 50% CerS2 null BM cells.

As DSS‐induced colitis is driven by innate immune cells 29, 30, we generated BM chimeras in which WT or CerS2 null mice received either CerS2 deficient or WT BM to examine whether any cells of haematopoietic origin are involved in the development of colitis in CerS2 null mice. We reduced the amount of DSS administered to the mice from 3% to 2% (w/v) to reduce mortality, and DSS was administered in the drinking water for 7 days to induce acute colitis in BM chimeric mice. Irrespective of the reconstituted BM types, CerS2 null recipients lost more weight, had significantly higher DAI scores and showed shorter colon lengths than WT recipients (Fig. 3A). We further generated mixed BM chimeras in which WT or CerS2 null mice received 50% CerS2 null and 50% WT BM. Similar to the data from BM chimeric mice, CerS2 null recipients with mixed BM also showed severe colitis (Fig. 3B). Although we cannot exclude completely the role of the immune system in this model, these data suggest that host environment (including epithelial cells in the colon) likely plays a major role under these conditions.

**Figure 4:**
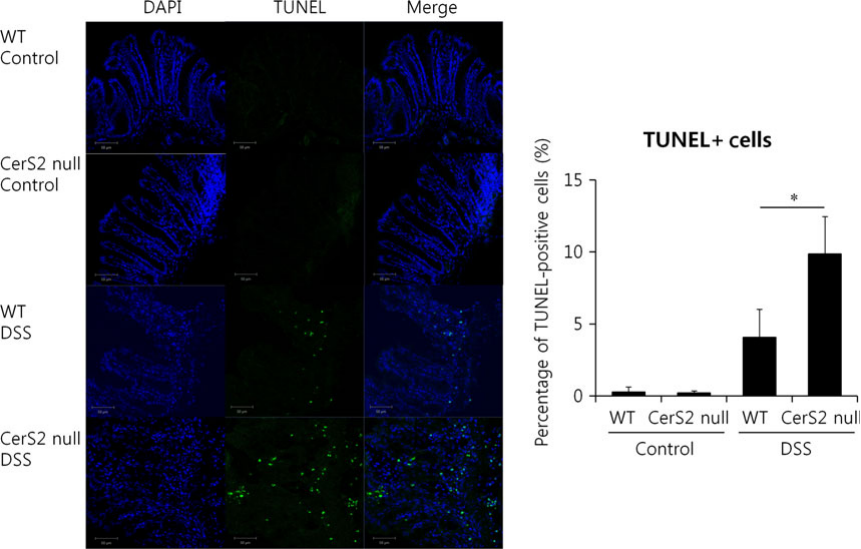
Increased apoptosis in CerS2 null mice with colitis. Mice were exposed to 2% (w/v) DSS in the drinking water for 7 days to induce colitis or normal plain water (control). TUNEL‐positive (green) cells indicating apoptotic cells were examined using immunofluorescence (left panel) and quantified using Image J (right panel); nuclei are counterstained with DAPI (blue) (400× magnification). Results are means ± S.E.M. (*n* = 4). *P < 0.05. Images are typical of four independent experiments.

**Figure 5:**
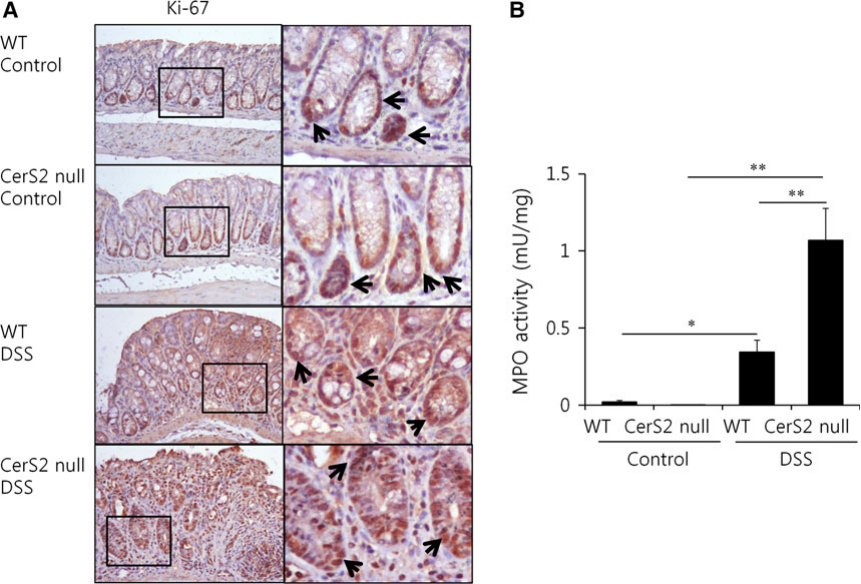
Increased myeloperoxidase activity in CerS2 null mice with colitis. Mice were exposed to 2% (w/v) DSS in the drinking water for 7 days to induce colitis or normal plain water (control). (**A**) Ki‐67‐positive cells undergoing proliferation are stained DAB as a chromogen (200× magnification). Arrows indicated Ki‐67‐positive cells. (**B**) Myeloperoxidase (MPO) enzymatic activity was determined as an index of neutrophil infiltration into injured colon tissue. Results are means ± S.E.M. (*n* = 5). *P < 0.05, **P < 0.01. Images are typical of three independent experiments.

**Figure 6:**
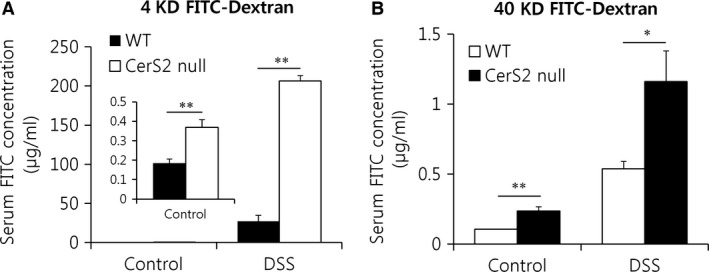
Intestinal permeability is increased in Cers2 null mice. Mice were exposed to 2% (w/v) DSS in the drinking water for 7 days to induce colitis or normal plain water (control). Mice were orally administered 4 (**A**) or 40 (**B**) kDa FITC‐dextran (0.6 g/kg bodyweight) in 0.1 ml phosphate‐buffered saline and fluorescence intensity in the serum was evaluated by spectrophotometry after 4 hrs. Results are expressed as means ± S.E.M. (*n* = 5). *P < 0.05, **P < 0.01.

Thus, we next evaluated the proliferation and death of non‐haematopoietic cells in the colon after DSS administration. TUNEL staining indicated that DSS treatment increased apoptosis in the colon of CerS2 null mice (Fig. 4), whereas cell proliferation was unaffected (Fig. 5A). Neutrophil infiltration, as determined by myeloperoxidase assay 31, was increased in the colon of CerS2 null mice treated with DSS (Fig. 5B). As defective epithelial barrier function has been implicated in IBD 32, we then evaluated intestinal permeability using FITC‐dextran (Fig. 6A and B). Compared to WT controls, blood levels of orally administered 4 or 40 kD FITC‐dextran were increased significantly in CerS2 null mice with and without DSS treatment, indicating increased intestinal permeability in CerS2 null mice under both basal and inflammatory states.

### Decreased JAM‐A expression in the colon of CerS2 null mice

Mucin dysfunction 33, 34 and JAM‐A deficiency 35, 36 have been shown to cause increased intestinal permeability, especially under basal conditions, with an enhanced susceptibility to experimental colitis. Thus, we evaluated mucin production in CerS2 null mice. The mucus blanket overlying the intestinal epithelium consists of an inner stratified layer that tightly adheres to epithelial cells and an outer unattached layer 33. These two layers are organized into a net‐like polymer around mucin 2, the most abundant mucin protein, which is heavily glycosylated and secreted by goblet cells 33. Mucin can be detected by PAS and alcian blue staining (PAS detects neutral carbohydrates, whereas alcian blue recognizes acidic carbohydrates that represent sialylated, fucosylated or sulphated sugars 34). However, PAS and alcian blue staining were normal in the colon of CerS2 null mice (Fig. S3), indicating normal mucin production.

**Figure 7:**
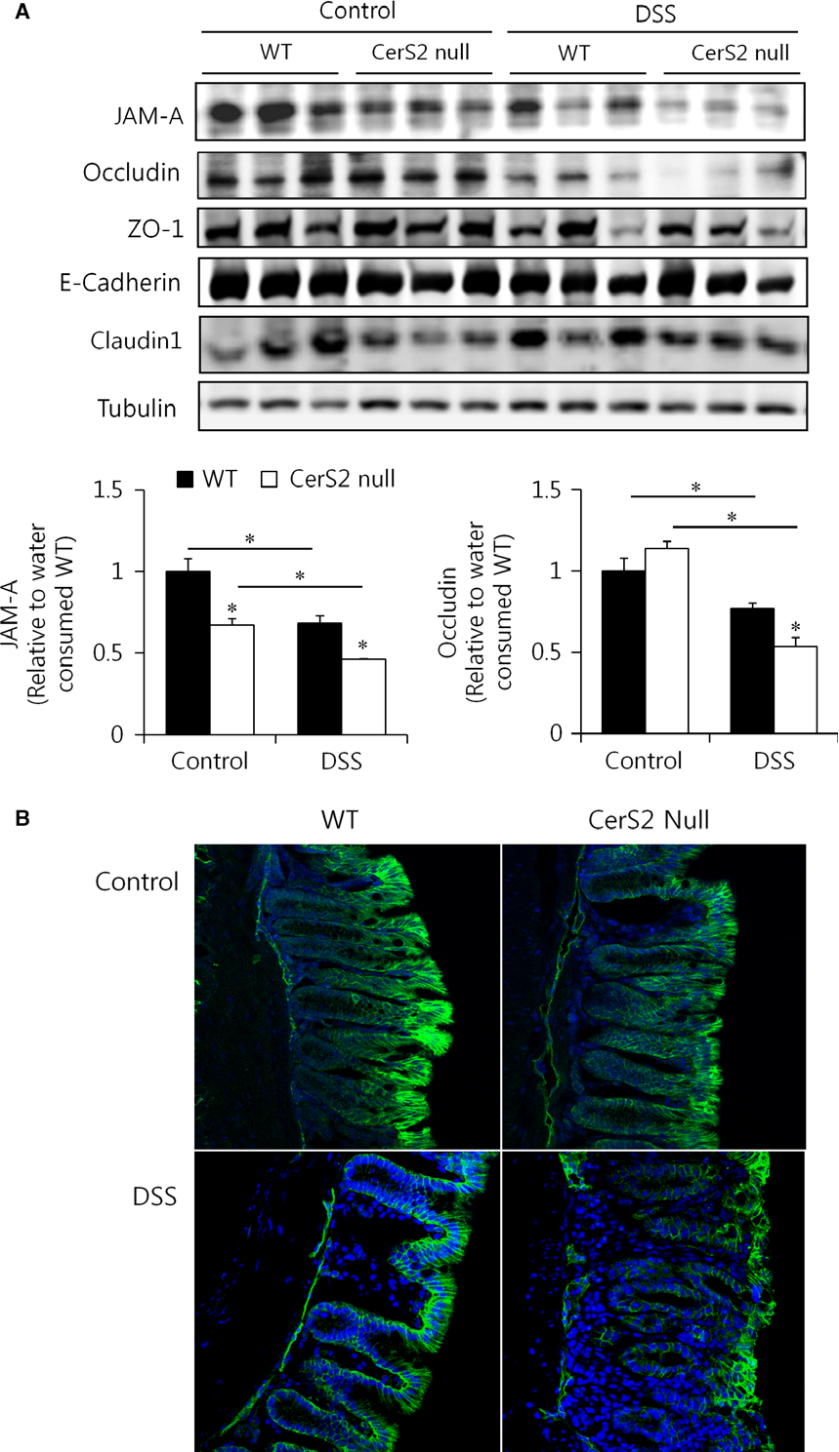
JAM‐A protein expression is diminished in the colon of CerS2 null mice. Mice were exposed to 2% (w/v) DSS in the drinking water for 7 days to induce colitis or normal plain water (control). (**A**) Representative Western blots of apical junctional complex proteins (upper panels) and quantification (lower panels) (*n* = 5) of JAM‐A and occludin in colon. *P < 0.05. (**B**) JAM‐A expression (green) in the colon upon DSS treatment in WT and CerS2 null mice was examined using immunofluorescence; nuclei are counterstained with DAPI (blue) (200× magnification). ZO‐1, zonula occludens‐1.

We next evaluated the expression of components of epithelial tight junctions. The epithelial cells lining the gastrointestinal tract are connected *via* desmosomes and gap junctions, as well as tight and adherens junctions 37, 38, which comprise the apical junctional complex that is generally believed to play an essential role in maintaining the epithelial barrier 37. In apical junctional complexes in CerS2 null mice, the expression of JAM‐A, which has been implicated in the regulation of barrier function and leucocyte migration 35, was significantly reduced with and without DSS treatment compared to WT mice (Fig. 7A and B). Although basal levels of occludin expression were not different between the colon of CerS2 null and WT mice, the expression decreased dramatically in CerS2 null mice treated with DSS (Fig. 7A). The levels of other junctional proteins, such as ZO‐1, E‐cadherin and claudins, were not altered significantly in CerS2 null mice compared to WT mice (Fig. 7A, Fig.S4).

### Increased MLC2 phosphorylation in the colon of CerS2 null mice

**Figure 8:**
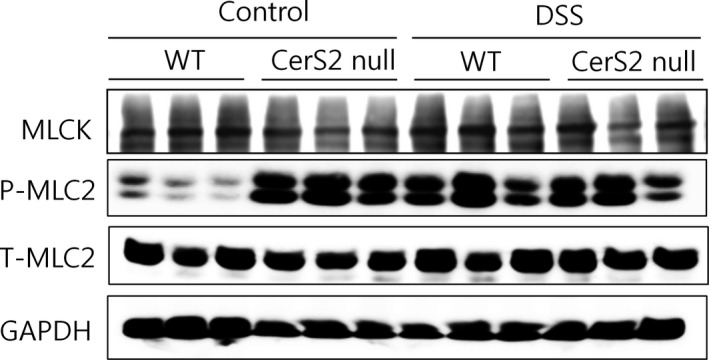
MLC‐2 phosphorylation is increased in colons of CerS2 null mice. Mice were exposed to 2% (w/v) DSS in the drinking water for 7 days to induce colitis or normal plain water (control). Representative Western blots of myosin light chain kinase (MLCK), phospho‐myosin light chain 2 (P‐MLC2), total‐MLC2 (T‐MLC2) and glyceraldehyde 3‐phosphate dehydrogenase (GAPDH). Data represent three independent experiments.

Myosin light chain kinase (MLCK)‐induced contractility of the perijunctional actomyosin ring, that encircles the enterocyte at the level of the tight junction through numerous smaller proteins 39, is considered another important mechanism underlying disruption of the epithelial barrier 37. Thus, we evaluated the expression of MLCK in colons of DSS‐treated and control mice. Protein expression was not altered in CerS2 null mice; however, MLC2 phosphorylation was increased significantly in the colon of CerS2 null mice (Fig. 8), suggesting that increased contraction of the actomyosin belt may contribute to the elevated intestinal permeability in these mice.

### CerS2 knockdown Caco‐2 cells display impaired barrier function

**Figure 9:**
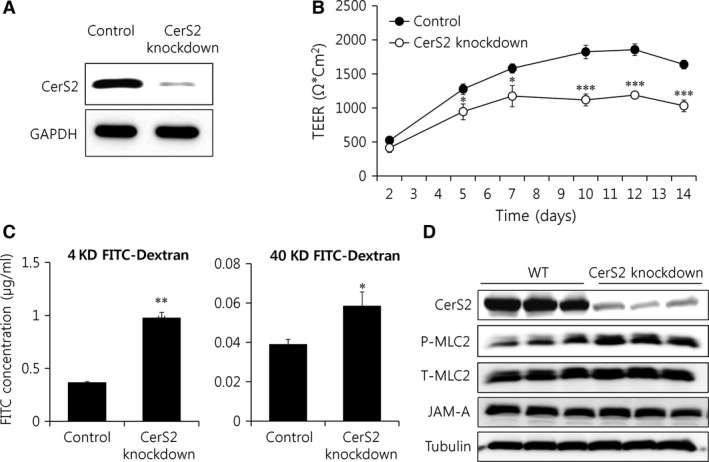
CerS2 deficiency leads to impaired cellular barrier function. CerS2‐knockdown Caco‐2 cells. (**A**) Representative Western blots of CerS2 and GAPDH. (**B**) TEER values from Caco‐2 cells were measured during spontaneous differentiation. (**C**) Permeability of differentiated Caco‐2 cells was analysed using 4 kDa (left panel) and 40 kDa (right panel) FITC‐dextran. (**D**) Representative Western blots of MLC2 phosphorylation and JAM‐A. Results are expressed as means ± S.E.M. (*n* = 3). *P < 0.05, **P < 0.01, ***P < 0.001. Data represents three independent experiments.

To confirm an important role of CerS2 in regulating intestinal barrier function, we used Caco‐2 cells, a human colon epithelial cell line, which differentiate spontaneously into a monolayer of polarized cells coupled by tight junctions after 2–3 week 40. We generated CerS2‐knockdown Caco‐2 cells using CRISPR‐Cas9 (Fig. 9A) and measured transepithelial electrical resistance (TEER) upon spontaneous differentiation. TEER is a widely accepted quantitative technique for measuring the integrity of tight junction dynamics in cultured endothelial and epithelial monolayers 41. Similar to the increased intestinal permeability in CerS2 null mice, CerS2 knockdown Caco‐2 cells displayed lower TEER levels during differentiation (Fig. 9B), confirming an important role of CerS2 in maintaining the integrity of the cellular barrier. In addition, CerS2 knockdown Caco‐2 cells also exhibited increased permeability to 4 and 40 kD FITC‐Dextran (Fig. 9C), as well as increased MLC2 phosphorylation (Fig. 9D). However, the levels of JAM‐A were not altered by CerS2 knockdown in these cells (Fig. 9D), suggesting that JAM‐A expression is not directly affected by CerS2 modulation. SL levels in CerS2 knockdown CaCo‐2 cells were altered similarly with the colon of CerS2 null mice (Fig. S5); levels of sphinganine and C16‐ceramides were increased with decrease of C22‐C24 ceramides.

### SL homeostasis is important for cellular barrier integrity

**Figure 10:**
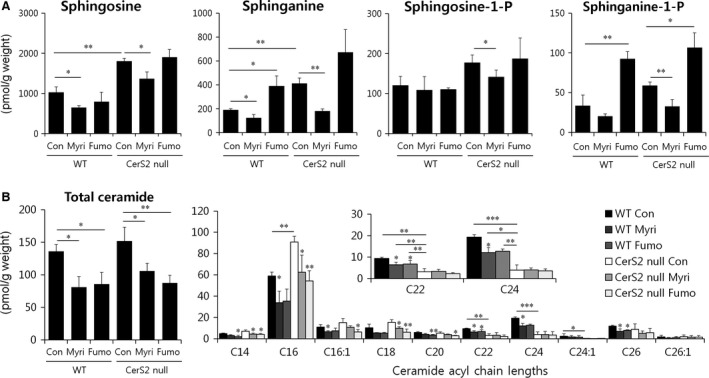
Effects of myriocin and fumonisin B1 administrations on colon sphingolipid levels. Mice were orally administered fumonisin B1 (1.5 mg/kg/day) or myriocin (0.5 mg/kg/day) for 10 days. Long‐chain bases (**A**) and ceramides (**B**) were measured by ESI‐MS/MS. The *x* axis of the right panel in **B** shows the acyl chain lengths of the individual ceramide species. Results are expressed as means ± S.E.M. (*n* = 3). *P < 0.05, **P < 0.01, ***P < 0.001. Con, control; Myri, myriocin; Fumo, fumonisin B1.

**Figure 11:**
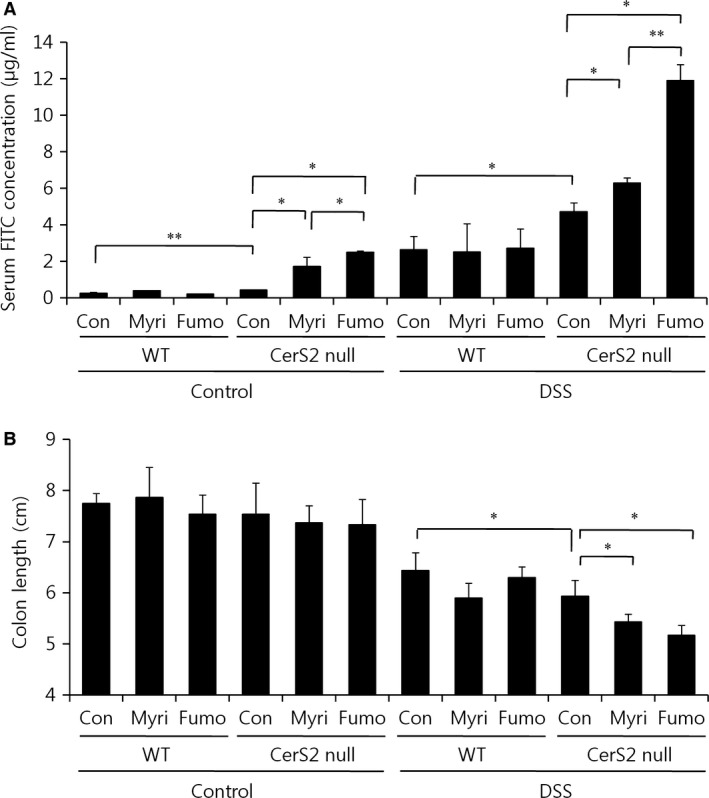
Administrations of fumonisin B1 and myriocin in Cers2 null mice increase intestinal permeability. Mice were orally administered fumonisin B1 (1.5 mg/kg/day) or myriocin (0.5 mg/kg/day) for 3 days and then exposed to 2% (w/v) DSS in the drinking water for 7 days to induce colitis with coadministration with fumonisin B1 or myriocin. (**A**) Mice were orally administered 4 kDa FITC‐dextran (0.6 g/kg bodyweight) in 0.1 ml phosphate‐buffered saline, and fluorescence intensity in serum was then examined by spectrophotometry after 4 hrs. (**B**) Colon lengths of mice were measured. Results are expressed as means ± S.E.M. (*n* = 4). *P < 0.05, **P < 0.01. Con, control; Myri, myriocin; Fumo, fumonisin B1.

Finally, we explored whether decreasing the levels of long‐chain bases and C16‐ceramide restores barrier integrity in colons of CerS2 null mice using oral fumonisin B1 and myriocin administration. Treatment of myriocin, a serine palmitoyltransferase inhibitor, decreased levels of SLs including long‐chain bases and C16‐ceramide as reported previously 42 (Fig. 10A and B). Treatment of fumonisin B1, a CerS inhibitor, led to elevation of long‐chain bases and decrease of ceramides including C16‐ceramide in accordance with previous reports 43, 44 (Fig. 10A and B). Myriocin administration in CerS2 null mice normalized the elevated levels of long‐chain bases and C16‐ceramides (Fig. 10A and B). Fumonisin B1 treatment in CerS2 null mice only normalized the elevated levels of C16‐ceramides, but not the levels of long‐chain bases (Fig. 10A and B). Both treatments aggravated the intestinal permeability of CerS2 null mice as measured by serum FITC‐dextran levels (Fig. 11A) and diminished the colon length of CerS2 null mice upon DSS administration (Fig. 11B), suggesting that elevated levels of long‐chain bases and C16‐ceramide themselves are not mainly responsible for the increased intestinal permeability in CerS2 null mice, and rather altered SL levels including reduction of C22‐C24‐ceramides may indeed play a crucial role in increased intestinal permeability of CerS2 null mice. However, fumonisin B1 administration increased intestinal permeability of CerS2 null mice much higher than myriocin administration, suggesting an aggravating role of long‐chain bases in intestinal permeability of CerS2 null mice. Although both myriocin and fumonisin B1 treatments in WT mice diminished total ceramide levels significantly, the levels of C22‐C24 ceramides in WT were still higher than that in CerS2 null mice (Fig. 10B). In addition, both treatments did not affect the intestinal permeability of WT mice as measured by serum FITC‐dextran levels (Fig. 11A) and the colon length of WT mice upon DSS administration (Fig. 11B), implicating that partially diminished levels of C22‐C24 ceramides by chemical administration are not sufficient to aggravate colitis symptoms.

## Discussion

The results from this study demonstrate that CerS2, which regulates levels of C22–C24‐SLs, plays an important role in maintaining intestinal epithelial barrier function. CerS2 null mice displayed increased intestinal permeability and exacerbated colitis induced by DSS. Although the pathogenesis of IBD remains unclear, increasing evidence suggests that intestinal permeability plays a crucial role, and defective epithelial barrier function has been reported to predict relapse during clinical remission 45. Thus, elucidating the mechanisms for defects in barrier function and permeability has a great potential for defining IBD pathogenesis and uncovering novel therapeutic targets.

Important roles of ceramides in maintaining intestinal barrier function have been suggested by several studies. For example, the accumulation of ceramide at the sites of cell/cell‐contact induced by exogenous sphingomyelinase increases intestinal permeability 46, and blocking ceramide generation using a sphingomyelinase inhibitor alleviates murine colitis 47. Although a specific increase of C18:1‐ceramides *via* alkaline ceramidase 3 deficiency exacerbates inflammation in experimental colitis 48, distinct roles of ceramides with other acyl chain lengths in IBD have not been explored. CerS2 generates C22‐C24 ceramides distinctly, and lack of CerS2 leads to a decrease of C22‐C24 ceramides with an increase of C16‐ceramides and long‐chain bases 23. As increased levels of ceramide have been reported to be associated with increased intestinal permeability 46, elevated levels of C16‐ceramides may cause increased intestinal permeability of CerS2 null mice. However, myriocin treatment which normalized levels of C16‐ceramides and long‐chain bases in the colon of CerS2 null mice even elevated intestinal permeability. The accumulation of ceramide in Caco‐2 cells by exogenous sphingomyelinase leads to an altered lipid composition of microdomain 46. Recently, we also reported that the acyl chain length of SLs determines the biophysical properties of membranes and affects lipid raft microdomain formation and insulin signalling in the liver 19. In addition, tight junction proteins including ZO‐1, occludin and JAM‐A are localized in lipid rafts, and lipid raft alteration induced by cholesterol depletion disrupts barrier function by altering the distribution of tight‐junction proteins 49. Therefore, altered membrane properties including lipid raft microdomain either by deficiency of CerS2 or by treatment of exogenous sphingomyelinase 46 may affect tight junction function in the intestine. Furthermore, membranes from CerS2 null mice exhibited a higher membrane fluidity 23 and increased membrane fluidity may influence intestinal permeability directly as modulation of permeability *via* altered membrane fluidity has been suggested by several studies 50, 51.

Both the colon of CerS2 null mice and CerS2 knockdown Caco‐2 cells displayed increased MLC2 phosphorylation, implicating CerS2 in apical actomyosin contraction. As MLCK protein expression was not increased in the colon of CerS2 null mice, CerS2 depletion may lead to increased MLC2 phosphorylation possibly *via* regulating MLCK or MLC2 phosphatase activity. Similar to CerS2 null mice, transgenic mice expressing constantly active MLCK also showed increased MLC2 phosphorylation with elevated intestinal permeability, but no overt histologic signs of intestinal inflammation 52. Consistent with the presence of increased permeability in some healthy first‐degree relatives of Crohn's disease patients 53, increased intestinal permeability of CerS2 null mice was also insufficient to cause spontaneous colitis or histologic signs of intestinal inflammation in the absence of other stimuli. Phosphorylation of MLC2 leads to tight junction rearrangement, and reduction of intestinal barrier function, and CD4^+^CD45RB^hi^ adoptive transfer into transgenic mice expressing constantly active MLCK resulted in a much more severe colitis compared with control mice 52. In addition, the localization of tight junction proteins in the transgenic mice expressing constantly active MLCK was also intact similar to that in CerS2 null mice 52.

As with CerS2 null mice, mice deficient in JAM‐A exhibit increased basal intestinal permeability with normal epithelial architecture 35. JAM‐A regulates apical actomyosin contraction, and deficiencies lead to enhanced MLC2 phosphorylation 36. However, unlike in mice with JAM‐A deficiency 35, CerS2 null mice did not show increased epithelial proliferation and formation of large lymphoid aggregates. A possible explanation for this is that JAM‐A was only partially decreased in the colons of CerS2 null mice. Furthermore, experiments with CerS2 knockdown Caco‐2 cells indicated that JAM‐A is not directly regulated by CerS2 expression, and thus, the diminished JAM‐A protein levels in the colons of CerS2 null mice may not be directly related with CerS2 deficiency.

Fumonisin B1 elevates levels of long‐chain bases and decreases levels of all kinds of ceramides and their derivatives 43, and relatively high doses (>50 μM) have been reported to cause impaired barrier function in porcine intestinal epithelial cells 54. Similarly, fumonisin B1 administration dramatically increased intestinal permeability in CerS2 null mice. However, both fumonisin B1 and myriocin administration cause increased intestinal permeability of FITC‐dextran only in CerS2 null mice but not in WT mice, suggesting that altered long‐chain bases or reduced levels of ceramides and their derivatives by themselves are insufficient to impair pre‐formed epithelial barrier function *in vivo*, but may exacerbate pre‐existing barrier dysfunction. In other words, decreased levels of ceramides (including very‐long‐chain ceramides by CerS2 deficiency) may be able to inhibit epithelial barrier formation of complex protein–protein networks that mechanically link adjacent cells and seal the intercellular space, but may be insufficient to disrupt pre‐formed intestinal barrier function 55. Otherwise, the degree of reduction in the levels of very long‐chain ceramides may determine epithelial barrier function considering that both myriocin and fumonisin B1 treatments only partially diminished very long‐chain ceramides. The different roles of ceramides according to the degree of reduction were also reported previously 15, 20. CerS2 haplodeficiency partially decreased very long‐chain ceramides, but the phenotype of CerS2 haplodeficiency on triacylglycerol accumulation was opposite with that of CerS2 total deficiency 20, 56. Still, the precise effects of long‐chain bases and specific ceramides in intestinal barrier function should be further elucidated in the future, as systemic administration of fumonisin B1 and myriocin can affect many organs other than colon.

In conclusion, our data suggest that CerS2 and the levels of very long‐chain ceramides are crucial for intestinal barrier function. The results of this study also suggest that SL homeostasis including altered acyl chain composition may be involved in the pathogenesis of IBD by regulating intestinal permeability, and the development of techniques modulating acyl chain lengths of ceramides might be applied as a novel therapy for IBD.

## Conflicts of interest

None.

## Supporting information


**Appendix S1** Materials and methods
**Fig. S1** DSS‐induced colitis is exacerbated upon CerS2 deficiency
**Fig. S2** Haematological analysis of white blood cells in WT and CerS2 null mice
**Fig. S3** Mucin production and mucin 2 expression are not altered in colons of CerS2‐null mice
**Fig. S4** Claudin expression is not altered in colons of CerS2 null mice
**Fig. S5** Effects of CerS2 knockdown on sphingolipid levels in CaCo‐2 cells
**Table S1** Primers used for real time PCR
