# Supplementary material for: Ablation of ceramide synthase 2 exacerbates dextran sodium sulphate‐induced colitis in mice due to increased intestinal permeability
Source: J Cell Mol Med. 2017 Jul 12;21(12):3565–78. doi: 10.1111/jcmm.13267 (PMC5706577; doi:10.1111/jcmm.13267)
Supplement: Supplementary file 1 — Appendix S1 Materials and methods Fig. S1 DSS‐induced colitis is exacerbated upon CerS2 deficiency Fig. S2 Haematological analysis of white blood cells in WT and CerS2 null mice Fig. S3 Mucin production and mucin 2 expression are not altered in colons of CerS2‐null mice Fig. S4 Claudin expression is not altered in colons of CerS2 null mice Fig. S5 Effects of CerS2 knockdown on sphingolipid levels in CaCo‐2 cells Table S1 Primers used for real time PCR [file JCMM-21-3565-s001.docx]

**Supplementary materials and methods**

## *Materials*

DSS (MW, 36–50 kDa) was purchased from MP Biomedicals (Santa Ana, CA, USA). Anti-JAM-A (for mouse) and anti-E-cadherin antibodies were purchased from R&D Systems (Minneapolis, MN, USA), anti-JAM-A (for human) was from Santa Cruz biotechnology (Santa Cruz, CA, USA), anti-occludin and anti-ZO-1 were from Invitrogen (Carlsbad, CA, USA), anti-MLC2 and anti-phospho-MLC2 antibodies were from Cell Signaling Technology (Beverly, MA, USA), anti-GAPDH (glyceraldehyde 3-phosphate dehydrogenase) antibody was from Ab Frontier (Seoul, South Korea), anti-Ki67 antibody was from Abcam (Cambridge, UK), and anti-mouse and anti-rabbit horseradish peroxidase antibodies were from Jackson ImmunoReseasrch, Inc. (West Grove, PA, USA). All other materials were purchased from Sigma-Aldrich (St. Louis, MO, USA).

## *H & E staining*

Histological scores were measured on H & E-stained sections by an observer blind to the experimental conditions using a previously published system [1,2] as follows: for epithelial damage, 0 = none, 1 = minimal loss of goblet cells, 2 = extensive loss of goblet cells, 3 = loss of crypt cells and extensive loss of goblet cells, and 4 = extensive loss of crypt cells and goblet cells; and for infiltration, 0 = none, 1 = crypt base, 2 = muscularis mucosa, 3 = extensive muscularis mucosa and oedema, and 4 = submucosal. The histological index is the sum of each individual measure.

## *Generation of bone-marrow chimaeric mice*

CerS2-null mice and wild-type (WT) recipients were irradiated with a single dose of 1,000 cGy and were given antibiotics (ciprofloxacin 1.0 %) for 2 wk in their drinking water. One day after the irradiation, bone marrow (BM) was collected from the tibias and femurs of donor (CerS2-null and WT) mice. The non-red blood cells were counted using a haemocytometer and Türk’s solution (to delete erythrocytes). Irradiated mice were injected i.v. with 3 × 10^6^ BM cells. For generating mixed BM chimaeras, BM from C57BL/6 CD45.1^+^ WT × 129S4/SvJae CD45.2^+^ WT F1 mice were mixed equally (1:1) with BM derived from C57BL/6 × 129S4/SvJae CD45.2^+^ F1 CerS2-null mice. The chimaeras were treated with DSS (as described above) 8–10 wk post-BM transplantation.

## *Evaluation of intestinal permeability*

A FITC-dextran tracer (4 or 40 kDa) in 0.1 ml phosphate-buffered saline was administered by oral gavage (0.6 g/kg body weight) and blood was extracted from mice via heart puncture after 4 h to collect haemolysis-free serum. The fluorescence intensity of appropriately diluted serum was measured using a Biotek Synergy H1M microplate reader (excitation, 485 nm; emission, 535 nm) [3]. Mice were administered fumonisin B1 (1.5 mg/kg/d) or myriocin (0.5 mg/kg/d) for 3 d by oral gavage and then exposed to 2% (w/v) DSS in the drinking water for 7 d to induce colitis [4-6].

## *Western blotting*

Colon tissues and Caco-2 cells were lysed in RIPA buffer (50 mM Tris-Cl [pH 7.5], 150 mM NaCl, 1% Nonidet P-40, 0.5% sodium deoxycholate, and 0.1% SDS) containing protease and phosphatase inhibitors (Sigma-Aldrich), and protein levels were quantified using a protein assay dye reagent (Bio-Rad Laboratories, Hercules, CA, USA). Quantified proteins were separated in an SDS polyacrylamide gel and transferred to a nitrocellulose membrane (Bio-Rad Laboratories). After blocking with 5% bovine serum albumin (Sigma-Aldrich) for 1 h, the membranes were incubated in primary antibodies overnight at 4°C followed by an incubated with secondary antibodies for 1 h at room temperature. Band signals were detected by Image Quant LAS-4000 mini imager (GE Healthcare Life Sciences, Fuji, Japan) using ECL Western blotting detection reagents (Amersham Biosciences, Little Chalfont, UK).

## *Real-time PCR*

Total mRNA was extracted from colon tissues using an RNeasy mini kit (Qiagen, Valencia, CA, USA), and cDNA was synthesized from the mRNA using a Verso cDNA synthesis kit (Thermo Fisher Scientific, Waltham, MA, USA) according to the manufacturer’s protocol. Primers are described in Supplementary Table 1. Quantitative PCR was performed using SYBR green PCR master mix (Applied Biosystems, Warrington, UK) and an ABI PRISM 7500 sequence detection system (Applied Biosystems). Relative gene expression was calculated as 2*^−^*^ΔΔ^*^CT^* as described previously [7].

## *Immunostaining of the colon*

Mouse colon tissues were fixed in 4% (w/v) formaldehyde, embedded in paraffin, and sectioned at a 4-μm thickness. For immunohistochemistry, endogenous peroxidase activity was quenched via incubating with 0.3% (v/v) hydrogen peroxide and 0.5% (v/v) HCl in methanol. Tissue sections were then heated to 100°C in 10 mM citrate buffer (pH 6.0) to retrieve antigens and were preincubated with normal goat serum (10%) for 1 h. The slides were then serially incubated with anti-Ki67 and the HRP-conjugated secondary antibody. Peroxidase activity was measured using 3,3′-diaminobenzidine as the chromogen, and H & E staining was performed for counterstaining using standard methods. For immunofluorescence, deparaffinized slides were preincubated with normal goat serum (10%) for 1 h and immunostained with antibodies against JAM-A or MUC2, followed by Cy2- or Cy3-conjugated secondary antibodies (1:100; Jackson ImmunoResearch Laboratories, Inc., West Grove, PA).

## *TUNEL staining*

Colon tissues were fixed in 4% (w/v) formaldehyde, embedded and frozen in optimal cutting temperature compound (Sakura Finetech, Torrance, CA, USA), and cryosectioned at a 7-μm thickness. DNA strand breaks were then labelled by staining the slides using an *in situ* cell death detection kit with fluorescein (Roche, Basel, Switzerland) according to manufacturer’s instructions. TENEL positive cells were quantified using Image J.

## *Periodic acid-Schiff and alcian blue staining*

Colon tissue sections (4-μm thick) were stained using a periodic acid-Schiff (PAS) staining kit (Polysciences, Washington, PA, USA) according to the manufacturer’s protocol. Briefly, slides were serially immersed in 0.5% periodic acid in Schiff’s reagent solution and in 0.55% potassium metabisulfite solution. The slides were rinsed with tap water to allow the colour to develop. Finally, slides were immersed in acidified Harris haematoxylin solution for 30 s to stain the nuclei blue, and were then washed, dehydrated, and coverslipped.

For alcian blue staining, slides were serially immersed in a 3% acetic acid solution for 3 min and in an alcian blue solution for 30 min. The slides were immersed in nuclear fast red solution for 3 min and then washed, dehydrated, and coverslipped. Mucosubstance, nuclei, and cytoplasm were stained blue, pink to red, and pale pink, respectively.

## *Myeloperoxidase assay*

The myeloperoxidase assay was performed using a kit (CytoStore, Calgary, Alberta, Canada) according to the manufacturer’s protocol.

## *Complete blood counts*

Complete blood counts were performed by Pathovet Veterinary Diagnostic Services (Kfar Bilu, Israel) using a Siemens ADVIA 2120i haematology analyser (Siemens Healthcare Diagnostics, Erlangen, Germany).

## *Mass spectrometry of SLs*

The levels of SLs in colon tissues and CaCo-2 cells were measured by liquid chromatography electrospray ionisation tandem mass spectrometry (LC-ESI-MS/MS) as described previously [8] with some modifications. Briefly, extracted lipids were injected into a high-performance liquid chromatography (Agilent 1200 series; Agilent, CA, USA) and separated through a reverse phase Kinetex C18 column (2.1 × 50 mm; inner diameter, 2.6 μm) (Phenomenex, St. Louis, MO, USA). Then, the column effluent was introduced into an API 3200 triple quadruple mass spectrometer (ABCIEX, Toronto, Canada) and analysed using electrospray ionisation in positive mode with multiple-reaction monitoring with Analyst 1.4.2 software (Applied Biosystems).

**Supplementary Figures**

**
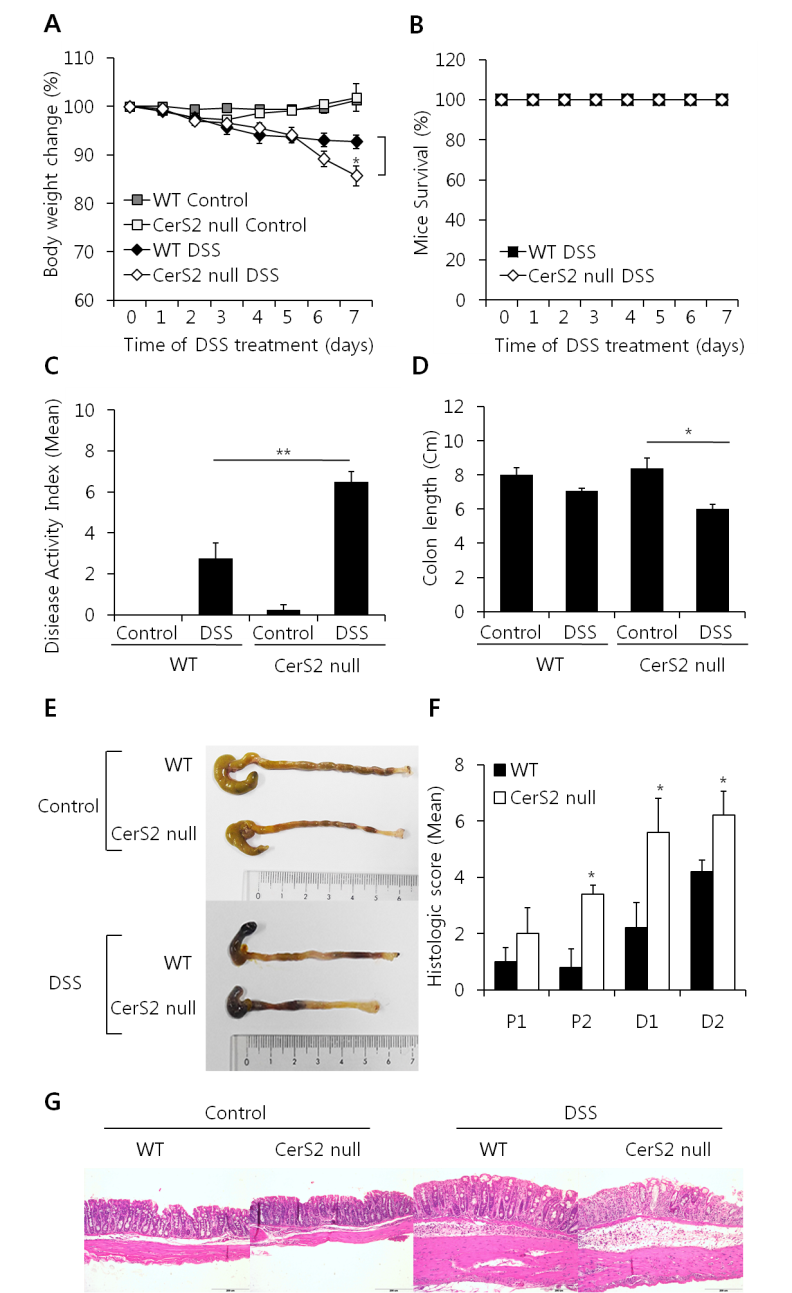
**

**Supplementary Fig. 1** DSS-induced colitis is exacerbated upon CerS2 deficiency. To induce colitis, mice were exposed to 2% (w/v) DSS in the drinking water for 7 d. For controls, normal plain water was consumed for 7 d. (**A**) Body weight changes and (**B**) survival curves of WT and CerS2 null mice. (**C**) Disease activity index values, (**D**) colon lengths, (**E**) representative colon pictures, (**F**) histological scores, and (**G**) H & E-stained colonic sections of mice. The scale bar indicates 200 µm. Data are means ± SEM (*n* = 4). **P* < 0.05, ***P* < 0.01.

**
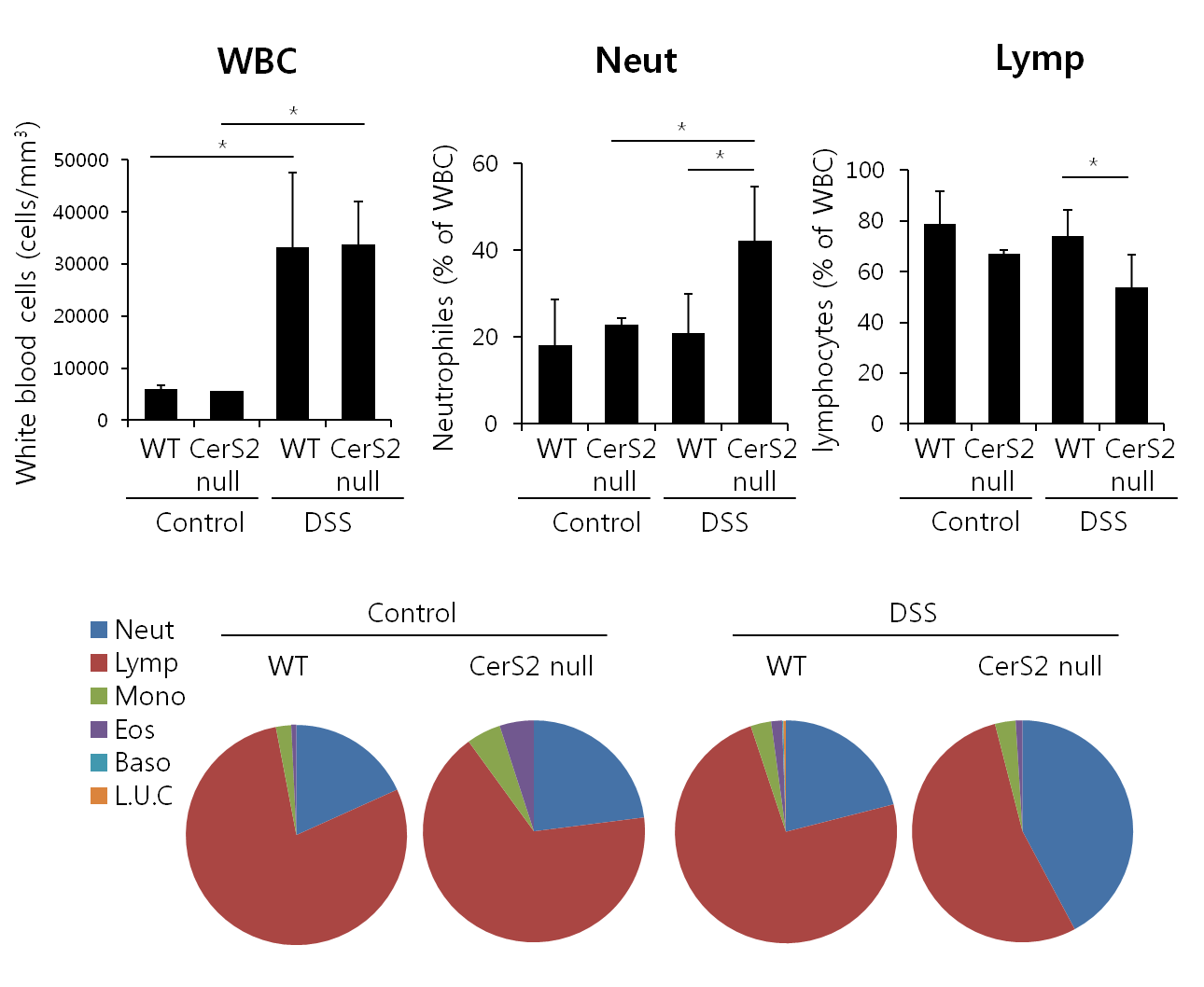
**

**Supplementary Fig. 2** Haematological analysis of white blood cells in WT and CerS2 null mice. Mice were exposed to 2% (w/v) DSS in the drinking water for 7 d to induce colitis or to normal plain water (control). The numbers and percentage compositions of white blood cells are demonstrated. Data are means ± SEM (*n* = 5). **P* < 0.05. WBC, white blood cells; Neut, neutrophil; Lymp, lymphocyte; Mono, monocyte; Eos, eosinophil; Baso, basophil; L.U.C., large unstained cells.

**
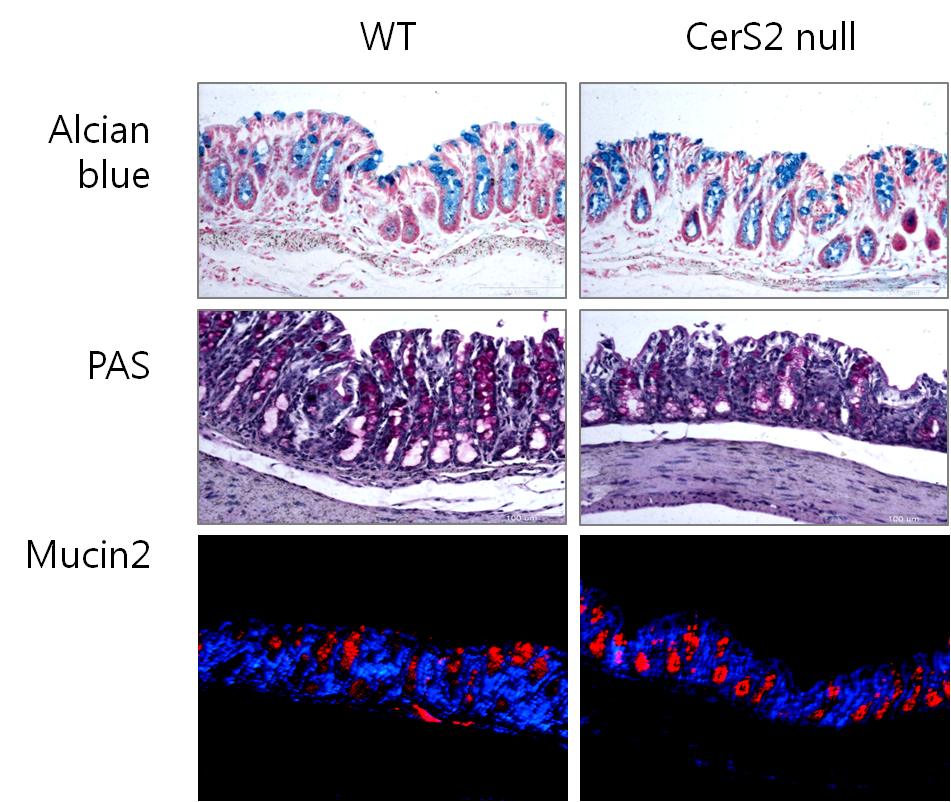
**

**Supplementary Fig. 3** Mucin production and mucin 2 expression are not altered in colons of CerS2-null mice. Mice were exposed to 2% (w/v) DSS in the drinking water for 7 days to induce colitis or normal plain water (control). Mucin was detected by alcian (top) and PAS (middle) staining. Image magnification, 200×. Mucin 2 expression (red; bottom) upon DSS treatment in colons of WT and CerS2-null mice was examined using immunofluorescence; nuclei are counterstained with DAPI (blue) (100× magnification). The image is a representative of three independent experiments.

**
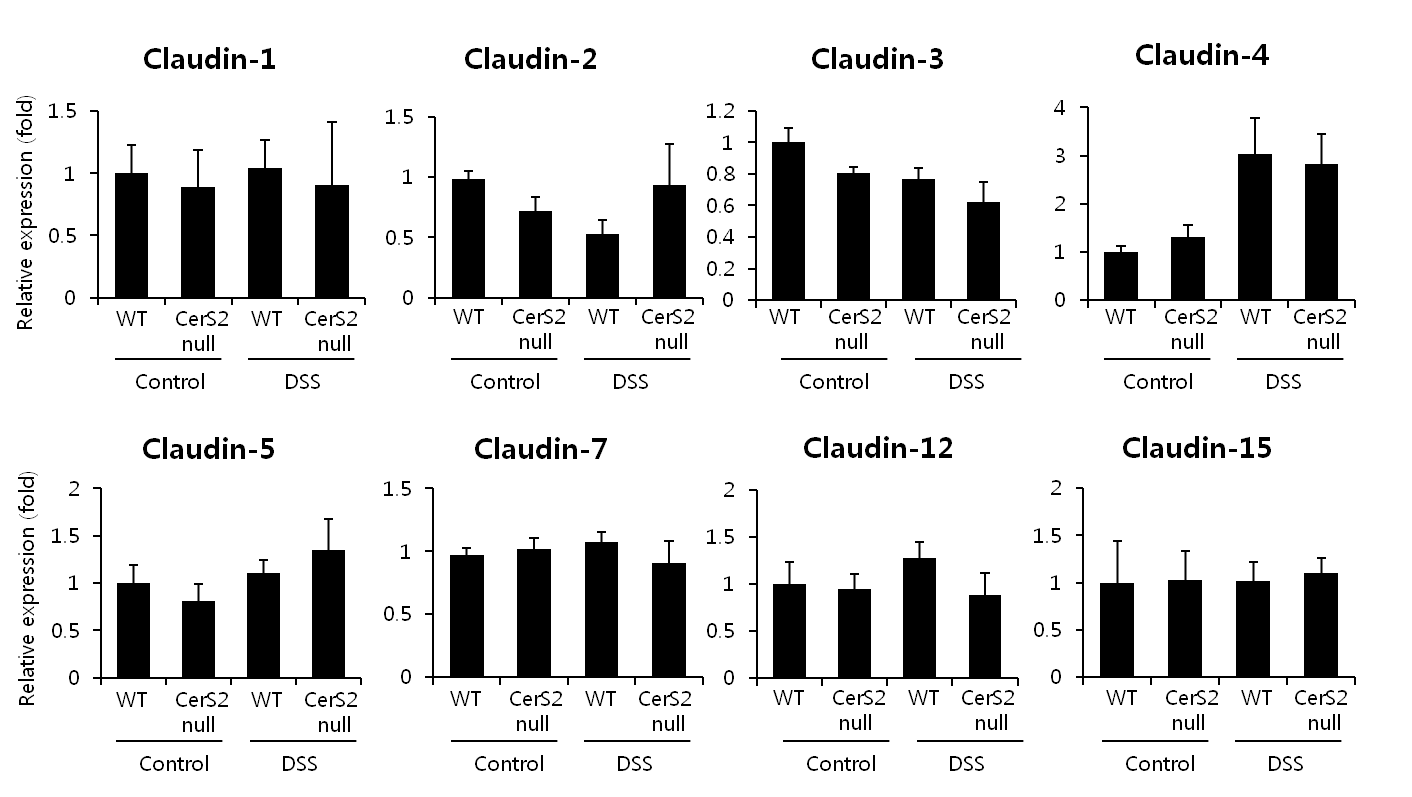
**

**Supplementary Fig. 4** Claudin expression is not altered in colons of CerS2 null mice. Mice were exposed to 2% (w/v) DSS in the drinking water for 7 days to induce colitis or normal plain water (control). Real-time polymerase chain reaction was performed to evaluate the transcription of claudin genes upon DSS treatment. Values are means ± SEM. (*n* = 5).


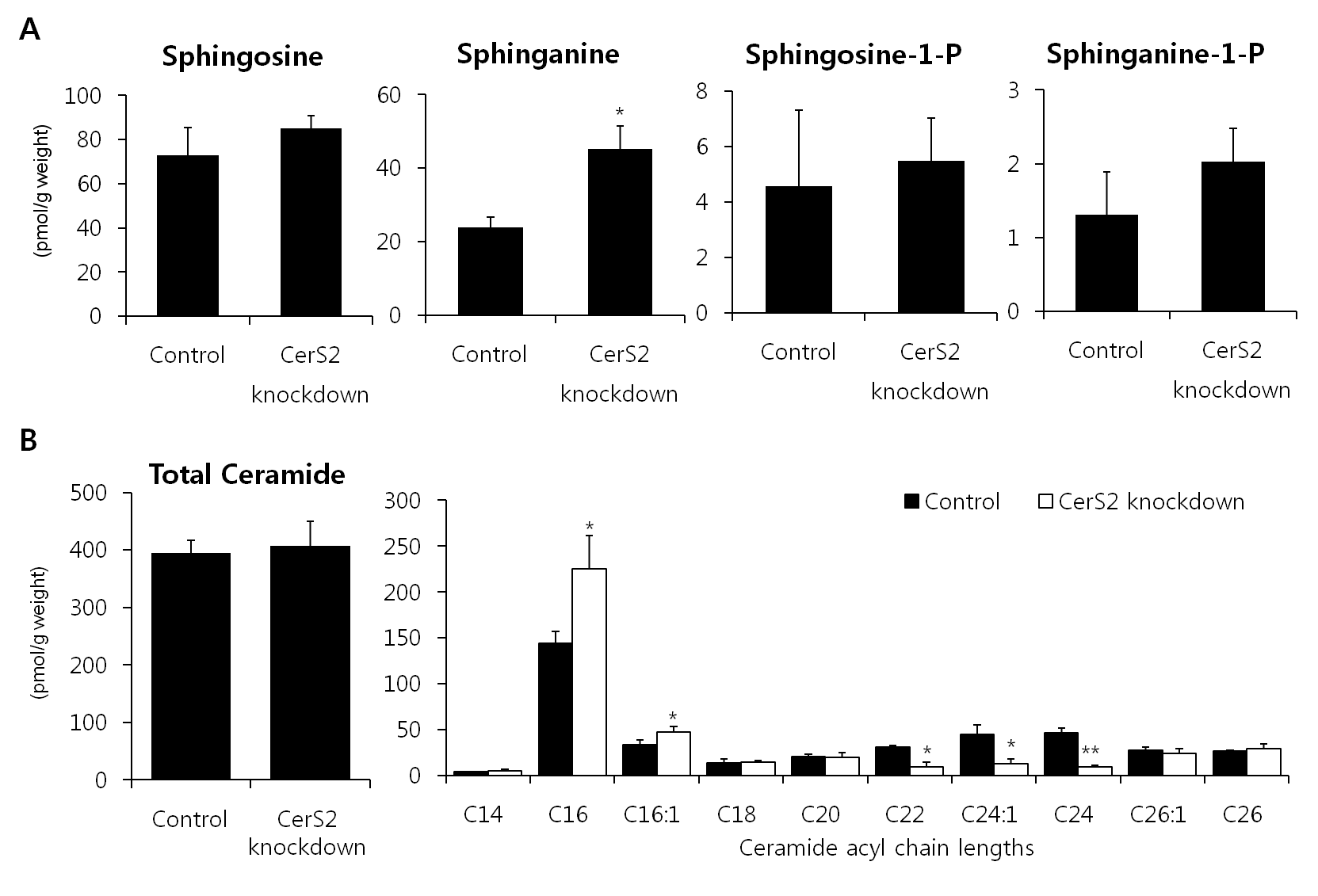


**Supplementary Fig. 5** Effects of CerS2 knockdown on sphingolipid levels in CaCo-2 cells. CerS2-knockdown Caco-2 cells were generated using CRISPR-Cas9 technology. Long-chain bases (**A**) and ceramides (**B**) were measured by ESI-MS/MS. The *x* axis of the right panel in **B** shows the acyl chain lengths of the individual ceramide species. Results are expressed as means ± SEM (*n* = 3). **P* < 0.05, ***P* < 0.01.

**Supplementary Table 1.** Primers used for real time PCR.

| Gene | Primer sequence (5’-3’) | References |
| --- | --- | --- |
| Claudin-1 | F: TCTACGAGGGACTGTGGATG | [9] |
|  | R: TCAGATTCAGCTAGGAGTCG |  |
| Claudin-2 | F: TATCTCTGTGGTGGGCATGA | [10] |
|  | R: CGAAGGATGCCATGAAGATT |  |
| Claudin-3 | F: GAGATGGGAGCTGGGTTGTA | [11] |
|  | R: GGATCTTGGTGGGTGCATAC |  |
| Claudin-4 | F: CGCTACTCTTGCCATTACG | [9] |
|  | R: ACTCAGCACACCATGACTTG |  |
| Claudin-5 | F: CTGGACCACAACATCGTGAC | [11] |
|  | R: GCCGGTCAAGGTAACAAAGA |  |
| Claudin-7 | F: AGCATGTTCCTGGATTGGTC | [12] |
|  | R: CCAGAAGGACCAGAGCAGAC |  |
| Claudin-12 | F: AACTGGCCAAGTGTCTGGTC | [11] |
|  | R: AGACCCCCTGAGCTAGCAAT |  |
| Claudin-15 | F: GATGGTGGCTATCTCGTGGT |  |
|  | R: GCACTCCAGCCCAAGTAGAG |  |
| Gapdh | F: CACTCTTCCACCTTCGATGC | [13] |
|  | R: CCCTGTTGCTGTAGCCGTAT |  |

**References**

1. **Cooper HS, Murthy SN, Shah RS, Sedergran DJ.** Clinicopathologic study of dextran sulfate sodium experimental murine colitis. Lab Invest. 1993 Aug;69(2):238-49.

2. **Martin GR, Keenan CM, Sharkey KA, Jirik FR.** Endogenous prion protein attenuates experimentally induced colitis. Am J Pathol. 2011 Nov;179(5):2290-301.

3. **Chassaing B, Aitken JD, Malleshappa M, Vijay-Kumar M.** Dextran sulfate sodium (DSS)-induced colitis in mice. Curr Protoc Immunol. 2014 Feb 04;104:Unit 15 25.

4. **Holland WL, Brozinick JT, Wang LP, et al.** Inhibition of ceramide synthesis ameliorates glucocorticoid-, saturated-fat-, and obesity-induced insulin resistance. Cell Metab. 2007 Mar;5(3):167-79.

5. **Park WJ, Park JW, Erez-Roman R, et al.** Protection of a ceramide synthase 2 null mouse from drug-induced liver injury: role of gap junction dysfunction and connexin 32 mislocalization. J Biol Chem. 2013 Oct 25;288(43):30904-16.

6. **Dugyala RR, Sharma RP, Tsunoda M, Riley RT.** Tumor necrosis factor-alpha as a contributor in fumonisin B1 toxicity. J Pharmacol Exp Ther. 1998 Apr;285(1):317-24.

7. **Pfaffl MW.** A new mathematical model for relative quantification in real-time RT-PCR. Nucleic Acids Res. 2001 May 01;29(9):e45.

8. **Shaner RL, Allegood JC, Park H, et al.** Quantitative analysis of sphingolipids for lipidomics using triple quadrupole and quadrupole linear ion trap mass spectrometers. J Lipid Res. 2009 Aug;50(8):1692-707.

9. **Bergmann KR, Liu SX, Tian R, et al.** Bifidobacteria stabilize claudins at tight junctions and prevent intestinal barrier dysfunction in mouse necrotizing enterocolitis. Am J Pathol. 2013 May;182(5):1595-606.

10. **Corridoni D, Pastorelli L, Mattioli B, et al.** Probiotic bacteria regulate intestinal epithelial permeability in experimental ileitis by a TNF-dependent mechanism. PLoS One. 2012;7(7):e42067.

11. **Weinl C, Castaneda Vega S, Riehle H, et al.** Endothelial depletion of murine SRF/MRTF provokes intracerebral hemorrhagic stroke. Proc Natl Acad Sci U S A. 2015 Aug 11;112(32):9914-9.

12. **Choi YS, Chakrabarti R, Escamilla-Hernandez R, Sinha S.** Elf5 conditional knockout mice reveal its role as a master regulator in mammary alveolar development: failure of Stat5 activation and functional differentiation in the absence of Elf5. Dev Biol. 2009 May 15;329(2):227-41.

13. **Park WJ, Kim SY, Kim YR, Park JW.** Bortezomib alleviates drug-induced liver injury by regulating CYP2E1 gene transcription. International Journal of Molecular Medicine. 2016 Mar;37(3):613-22.
